# Characterization of Four Type IV Pilin Homologues in *Stigmatella* *aurantiaca* DSM17044 by Heterologous Expression in *Myxococcus xanthus*

**DOI:** 10.1371/journal.pone.0075105

**Published:** 2013-09-18

**Authors:** Zaigao Tan, Haoming Li, Hongwei Pan, Xiuwen Zhou, Xin Liu, Ningning Luo, Wei Hu, Yuezhong Li

**Affiliations:** State Key Laboratory of Microbial Technology, School of Life Science, Shandong University, Jinan, Shandong, China; University of North Dakota School of Medicine and Health Sciences, United States of America

## Abstract

As prokaryotic models for multicellular development, *Stigmatella* *aurantiaca* and *Myxococcus xanthus* share many similarities in terms of social behaviors, such as gliding motility. Our current understanding of myxobacterial grouped-cell motilities comes mainly from the research on *M. xanthus*, which shows that filamentous type IV pili (TFP), composed of type IV pilin (also called PilA protein) subunits, are the key apparatus for social motility (S-motility). However, little is known about the pilin protein in *S* *. aurantiaca* . We cloned and sequenced four genes (*pilA*_Sa1~4_) from *S* *. aurantiaca* DSM17044 that are homologous to *pilA*_Mx_ (*pilA* gene in *M. xanthus* DK1622). The homology and similarities among PilA_Sa_ proteins and other myxobacterial homologues were systematically analyzed. To determine their potential biological functions, the four *pilA*_Sa_ genes were expressed in *M. xanthus* DK10410 (Δ*pilA*_Mx_), which did not restore S-motility on soft agar or EPS production to host cells. After further analysis of the motile behaviors in a methylcellulose solution, the *M. xanthus* strains were categorized into three types. YL6101, carrying *pilA*_Sa1_, and YL6104, carrying *pilA*_Sa4_, produced stable but unretractable surface pili; YL6102, carrying *pilA*_Sa2_, produced stable surface pili and exhibited reduced TFP-dependent motility in methylcellulose; YL6103, carrying *pilA*_Sa3_, produced unstable surface pili. Based on these findings, we propose that *pilA*_Sa2_ might be responsible for the type IV pilin production involved in group motility in *S* *. aurantiaca* DSM17044. After examining the developmental processes, it was suggested that the expression of PilA_Sa4_ protein might have positive effects on the fruiting body formation of *M. xanthus* DK10410 cells. Moreover, the formation of fruiting body in *M. xanthus* cells with stable exogenous TFP_Sa_ were compensated by mixing them with *S* *. aurantiaca* DSM17044 cells. Our results shed some light on the features and functions of type IV pilin homologues in *S* *. aurantiaca* .

## Introduction

Myxobacteria belong to a branch of intriguing prokaryotes recognized for their complex social behaviors [1]. A group of myxobacterial cells, including cells from *Myxococcus xanthus* and *Stigmatella* *aurantiaca* , can crawl in swarms on solid surfaces, cooperatively prey on environmental macromolecules or microbial cells, and accumulate at a center to form fruiting bodies when food is exhausted [2,3]. Our current understanding of myxobacterial social cell behaviors comes mainly from research on *M. xanthus*, which shows that social motility (S-motility) plays a fundamental role in these processes [4,5]. Three constituents, i.e., type four pili (TFP), extracellular polysaccharides (EPS) and lipopolysaccharide (LPS) O-antigens, are known to be essential for S-motility [5,6,7,8,9]. Among them, TFP act as molecular engines to enable S-motility, which are composed of thousands of protein subunits called type IV pilin (or the PilA protein) [6,10]. During S-motility, TFP function by extending at one of the cell poles, attaching to the solid surfaces of the substratum or another cell, and then retracting to pull the cell forward [10,11,12,13,14]. To achieve the cycles of extension and retraction, pilin proteins are assembled into polar filaments mediated by the ATPase PilB, and the extracellular TFP are disassembled into single subunits with the assistance of the ATPase PilT [13,15]. In addition to being the key apparatus for S-motility, TFP also play divergent roles in other physiological aspects of *M. xanthus*. Extracellular TFP provides proximity signals to the Dif chemosensory pathway to modulate EPS production [16], and the specific cellular pilin localization is required to maintain the normal amount of secreted EPS [17]. Moreover, the TFP apparatus has been proposed to be involved in plasmid natural transformation in *M. xanthus* [18].

*S* *. aurantiaca* and *M. xanthus* are both in the suborder Cystobacterineae of Myxococcales [1]. They appear very similar to each other in terms of social behaviors and both serve as prokaryotic models for multicellular development [19]. While the morphology of fruiting bodies varies, e.g., *M. xanthus* fruiting bodies are haystack-shaped and *S* *. aurantiaca* elaborate fruiting bodies that consist of tree-like stalks bearing several spore-filled sporangioles at their tops [1], the genetic programs for fruiting body formation and associated characteristics of the two species are very similar [20]. Unlike *M. xanthus*, relatively little is known about the motility in *S* *. aurantiaca* . *S* *. aurantiaca* and *M. xanthus* both require calcium ions for gliding [21], and inhibitors of protein synthesis prevent both the motility in *S* *. aurantiaca* and S-motility in *M. xanthus* [21]. Furthermore, energy-dependent cohesion and motility are suggested to be related phenomena in *S* *. aurantiaca* [21,22], which is consistent with the finding in *M. xanthus* that EPS is involved in both cohesion and S-motility [9,23]. Despite these known similarities between the motility in *S* *. aurantiaca* and *M. xanthus*, the features of the pilin protein, potentially the key component in grouped-cell motility, have not been investigated in *S* *. aurantiaca* *.*

Strain DSM17044 is the type strain of the *S* *. aurantiaca* species [24] and is closely related to another lab strain of *S* *. aurantiaca* , DW4/3-1. In this study, four genes homologous to the *pilA* gene in *M. xanthus* were cloned from *S* *. aurantiaca* DSM17044, and subsequently expressed in *M. xanthus* cells to characterize their products. The motility and development-related phenotypes of *M. xanthus* cells carrying different *S* *. aurantiaca* *pilA* homologues were systematically investigated. The results obtained in this study could help to understand the potential biological functions of the type IV pilin homologues in *S* *. aurantiaca* .

## Results

### Four genes in *S* *. aurantiaca* DSM17044 encode type IV pilin homologues

The genome of *S* *. aurantiaca* strain DW4/3-1 was recently sequenced [20], in which five genes were annotated as *pilA* homologues (the predicted product is a type IV pilus subunit or fimbrial protein), i.e., locus tag *STAUR*_*0004*, *1125*, *6449*, *6450* and *6924* (Genome access No. NC014623.1 in the GenBank database). Because strain DSM17044 is the type strain of the *S* *. aurantiaca* species [24] and is closely related to strain DW4/3-1, similar *pilA* homologues were expected to exist in strain DSM17044. Therefore, five sets of specific primers (listed in Table 1) were designed according to the sequences of the five *pilA* homologues in strain DW4/3-1, and four genes, *pilA*_Sa1_, *pilA*_Sa2_, *pilA*_Sa3_ and *pilA*_Sa4_ (see *Material sand Methods*), were amplified from DSM17044 genomic DNA with the primer sets targeting genes *STAUR_0004*, *6449*, *6450* and *6924* in the DW4/3-1 genome, respectively. Despite testing several different conditions, PCR using the primer pair Stig pilA-5-F and -R (Table 1) did not result in any specific products (data not shown).

**Table 1:** Primers used in this study.

**Primer**	**Sequence (5’~3’**)	**Description**
DK *pilA* SP-F	GTGAAGACCCGTGCTGCGGAGTTGC	Used in cloning *pilA*_Mx_ promoter and signal peptide (PSP_Mx_) sequence from *M. xanthus* DK1622 genomic DNA
DK *pilA* SP-R	GCCACGGTTGCGGGGGTTGAATC	
DK *pilA*-R	CGAGTTACTGGGCCGCGCCGTCG	Used to amplify PSP_Mx_-*pilA*_Mx_
Stig *pilA*-1-F	TTCAACCCCCGCAACCGTGGCTTTCACCCTCATCGAACTCATGATTG	Used in cloning *pilA*_Sa1_ gene from *S* *. aurantiaca* DSM17044 genomic DNA; designed according to sequence of *STAUR_0004* * in DW4/3-1 genome
Stig *pilA*-1-R	TTAGTCGCAGCTGACGTCGTTG	
Stig *pilA*-2-F	TTCAACCCCCGCAACCGTGGCTTCACCCTCATCGAGCTGATGATC	Used in cloning *pilA*_Sa3_ gene from *S* *. aurantiaca* DSM17044 genomic DNA; designed according to sequence of *STAUR_6449* * in DW4/3-1 genome
Stig *pilA*-2-R	TTACTGGCAGTTCACGTCGTTG	
Stig *pilA*-3-F	TTCAACCCCCGCAACCGTGGCTTTACGCTCATCGAGCTGATGATC	Used in cloning *pilA*_Sa4_ gene from *S* *. aurantiaca* DSM17044 genomic DNA; designed according to sequence of *STAUR_6450* * in DW4/3-1 genome
Stig *pilA*-3-R	CTACTCGCAGTCCACGTCATTGTT	
Stig *pilA*-4-F	TTCAACCCCCGCAACCGTGGCTTCACCCTCATTGAGCTCATGATT	Used in cloning *pilA*_Sa2_ gene from *S* *. aurantiaca* DSM17044 genomic DNA; designed according to sequence of *STAUR_6924* * in DW4/3-1 genome
Stig *pilA*-4-R	TTACGGGCAGTTGACGTCGTTG	
Stig *pilA*-5-F	TTCAACCCCCGCAACCGTGGCTTCACCTTTCTCGAAGTGTTGATC	Designed according to sequence of *STAUR_1125* * in DW4/3-1 genome
Stig *pilA*-5-R	TCAGAAGTCGCACTGGGTGTCCT	
RT-*pilA*_Sa1_-F	GCCAGCATCGCCATTCCGAGTTTCA	Used to investigate transcription of *pilA*_Sa1_ in DSM17044
RT-*pilA*_Sa1_-R	TCGTGCTGCGGTCCTCGTAAGAAGA	
RT-*pilA*_Sa2_-F	TCTGGCTTTACCCTCATCGAACTCA	Used to investigate transcription of *pilA*_Sa2_ in DSM17044
RT-*pilA*_Sa2_-R	AGATGCTGCAGTCTCCGAGGTGATA	
RT-*pilA*_Sa3_-F	TCGTGGTCGCCATCATCGGCATCCT	Used to investigate transcription of *pilA*_Sa3_ in DSM17044
RT-*pilA*_Sa3_-R	TCAGCGAGACCGTCGGGAAGTTACC	
RT-*pilA*_Sa4_-F	GGAGCCCCACAACGACGACAACT	Used to investigate transcription of *pilA*_Sa4_ in DSM17044
RT-*pilA*_Sa4_-R	AACCAGGTATCCGCCGTATCCGAGA	

* The locus tag of gene in *S* *. aurantiaca* DW4/3-1 genome.

**Figure 1:**
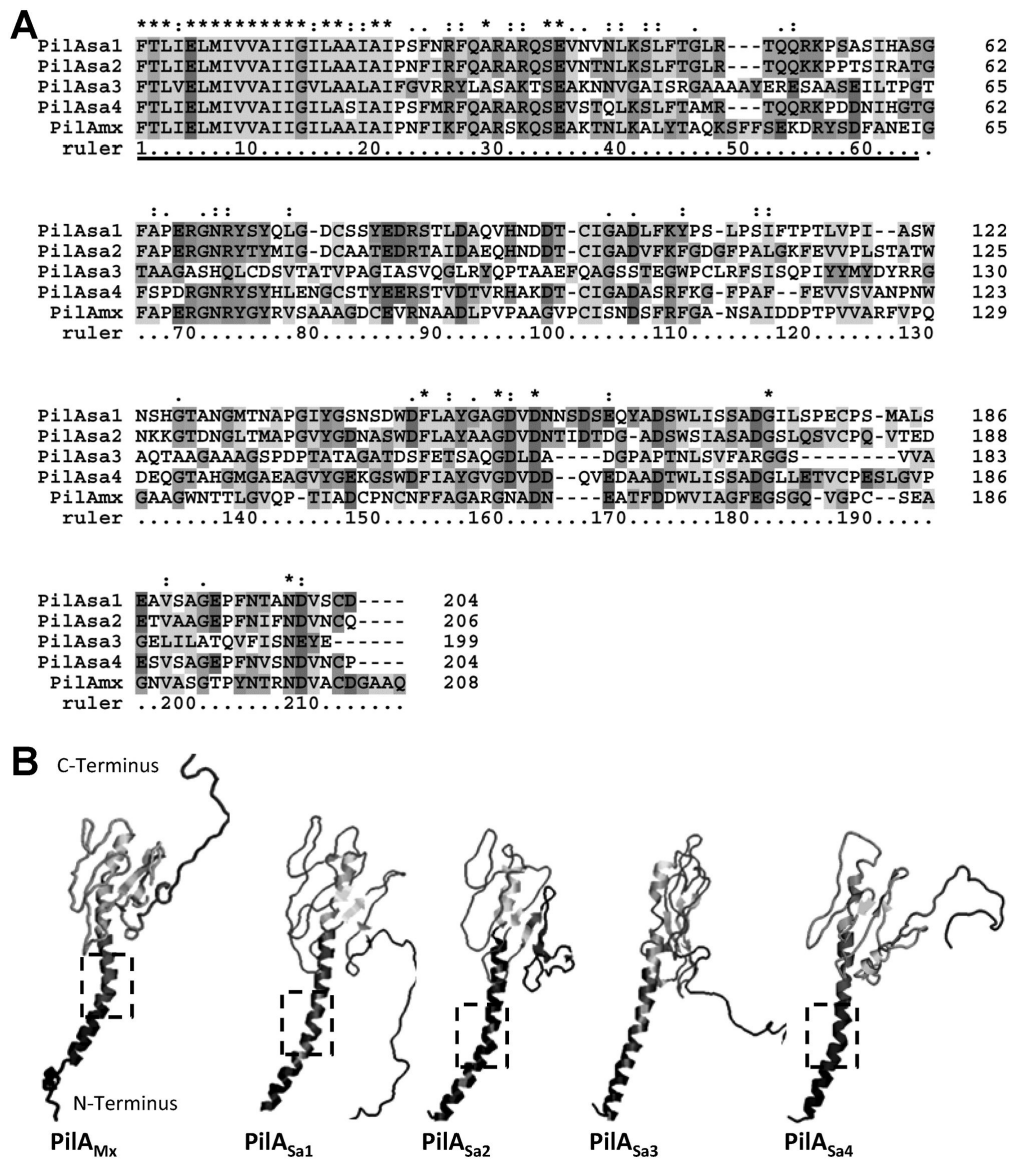
Four type IV pilin homologues in *S* *. aurantiaca* DSM17044. (A) Amino acid sequence alignment among type IV pilin in *M* *. xathus* DK1622 (PilA_Mx_) and the four homologues in *S* *. aurantiaca* DSM17044 (PilA_Sa1~4_). The underlined sequences correspond to the predicted N-terminal α-helical structures in panel B. (B) The 3D structures of the PilA_Mx_ and PilA_Sa1~4_ were predicted using 3D-JIGSAW and Swiss-model as described in the *Materials and Methods*. The dashed frames indicate the kink regions in α-N-terminal subdomains of the pilin structures.

After sequence alignment (Figure 1A), four PilA_Sa_ proteins from *S* *. aurantiaca* DSM17044 were found to share homology with the type IV pilin PilA_Mx_ from *M. xanthus* DK1622. In particular, the N-terminal sequences (1~43 residues) of the five proteins are well conserved, which is consistent with the finding that the first 28 residues of mature pilin are highly conserved among a variety of bacterial species [12,25,26]. Moreover, an N-terminal α-helix has been identified in all crystal structures of type IV pilins, e.g., PilA in *Pseudomonas aeruginosa* and PilE in *Neisseria gonorrhoeae* [25,26,27,28,29], which is packed in the filamentous TFP core [29]. As shown in Figure 1B, the simulated three-dimensional conformations of PilA_Mx_ and PilA_Sa_ proteins all exhibit spoon-like structures, in which the highly apolar N-terminal residues form an extended α-helical secondary structure. Interestingly, PilA_Mx_ and PilA_Sa1, 2, 4_ proteins all show a kink region in the α-helix while PilA_Sa3_ has an almost straight α-helical domain (Figure 1B), which may be due to the difference in their primary structures of residues 22~27 (Figure 1A).

In the alignment (Figure 1A), the C-terminal sequences of the five proteins are variable, and the low-score segments are mostly in PilA_Sa3_ protein sequence. In the putative structures (Figure 1B), the C-terminal globular domain were observed in all five proteins, which is believed to be exposed to the outer surface of TFP and involved in the biological functions of TFP [30,31]. It was also noticed that approximately 20 residues on the C-terminus of all five proteins exhibited random folding, which might be because this part of the sequence was missing in the models of the 3D structure prediction, e.g., PilA in *P. aeruginosa* and PilE in *N. gonorrhoeae*. Indeed, a previous study showed that the sequence of PilA_Mx_ was at least 17 residues longer than the pilin from *P. aeruginosa* or *N. gonorrhoeae* [12]. Despite the random folding portion, PilA_Mx_ and PilA_Sa1, 2, 4_ proteins were predicted to fold similarly at their C-terminal domains, while PilA_Sa3_ formed a more tightly packed C-terminal global structure compared to others.

**Figure 2:**
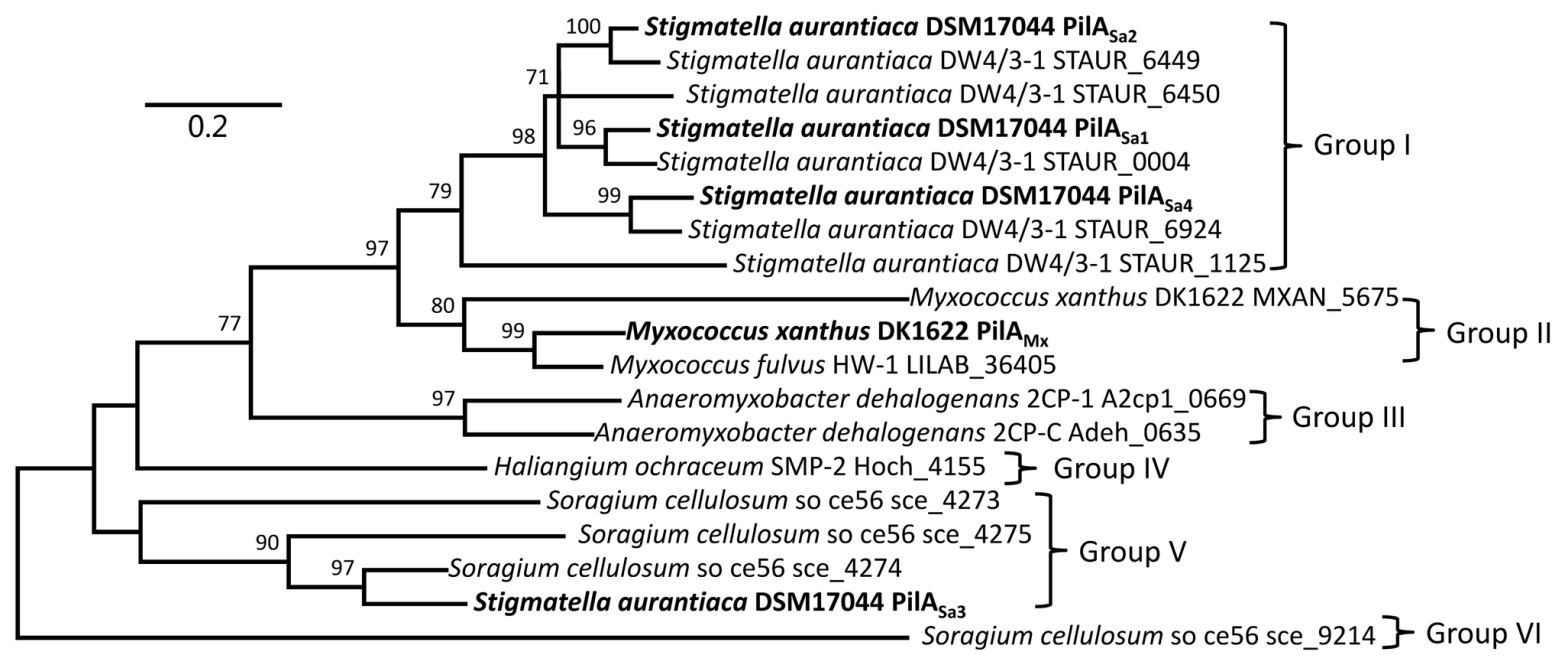
Phylogenetic analysis of the proteins homologous to type IV pilin from different myxobacterial strains. The bar indicated the evolutionary distance. The numbers on branch nodes were percentages of 1000 sets of bootstrap supports. The proteins, except for those from *S* *. aurantiaca* DSM17044, were denoted as their gene locus tags in the genome of the strain they belonged to.

Next, the similarities among PilA_Sa_ proteins and other myxobacterial homologues were further explored. The amino acid sequences of predicted pilin proteins from different myxobacterial strains were retrieved from the Genbank database and subjected to phylogenetic analysis. The strains belong to Cystobacterineae, Sorangineae and Nannocystineae suborders. As shown in Figure 2, 19 homologous PilA proteins from 8 strains could be divided into 6 deeply branched groups, and proteins from the same or closely related species tended to cluster together. As expected, PilA_Sa1, 2, 4_ from *S* *. aurantiaca* DSM17044 showed great similarities to proteins STAUR_0004, 6449 and 6924 from *S* *. aurantiaca* DW4/3-1, respectively, which is consistent with our initial primer design (Table 1). Surprisingly, PilA_Sa3_ is more similar to PilA proteins in *Sorangium cellulosum* so ce56 (e.g., SCE_4274) rather than its primer-targeted protein STAUR_6450 in *S* *. aurantiaca* DW4/3-1.

### Expression of four *pilA*_Sa_ genes in *M. xanthus* 10410 did not restore S-motility on agar or EPS production

**Figure 3:**
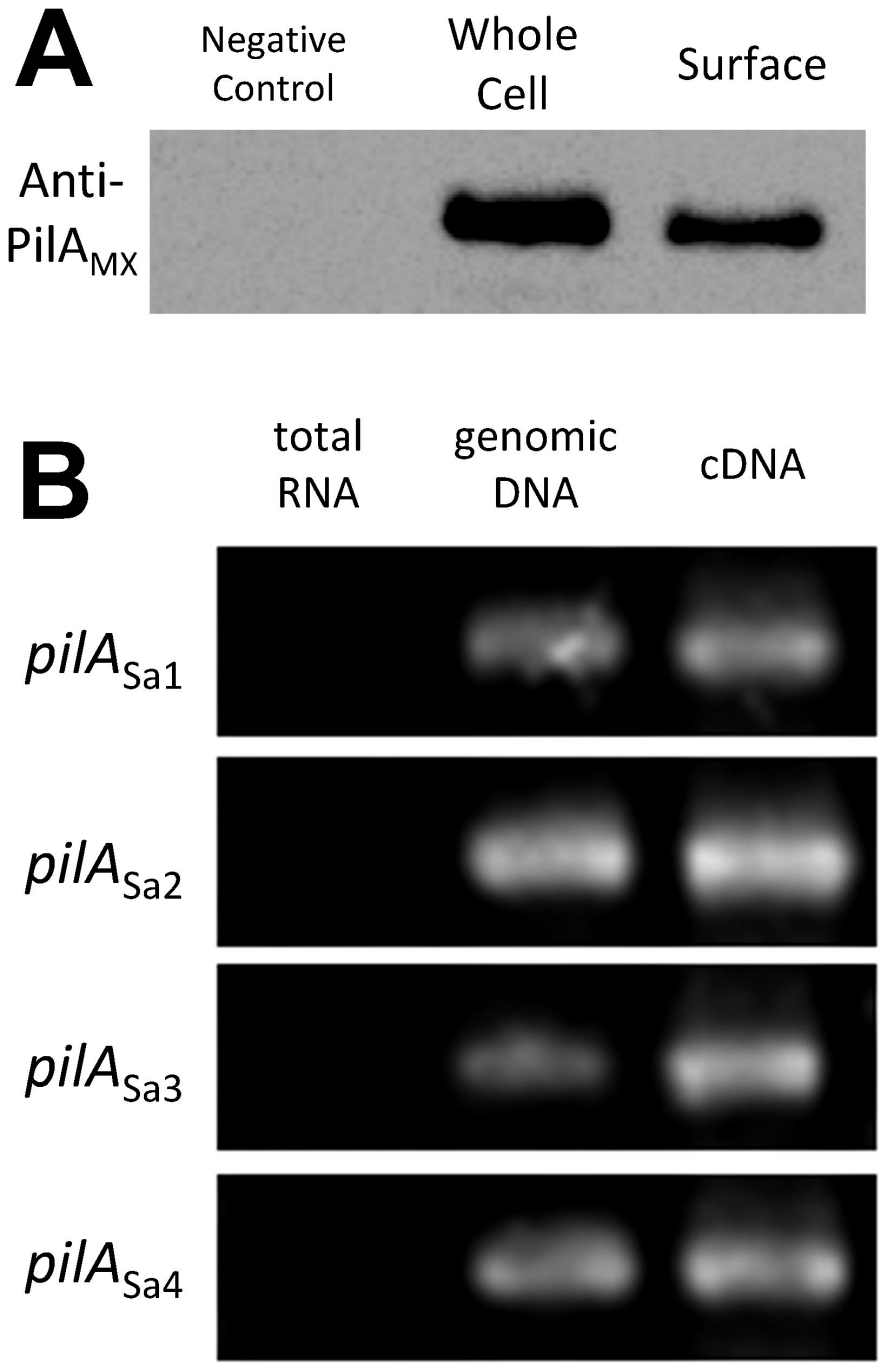
The expression and transcriptions of the *pilA*_Sa_ genes in *S* *. aurantiaca* DSM17044. (A) Whole-cell pilin (lane 2) and surface pili (lane 3) of *S* *. aurantiaca* DSM17044 cells were tested using western-blot probed by anti-PilA_Mx_ antibody. The whole-cell lysate of *M. xanthus* DK10410 (Δ*pilA*_Mx_) was loaded in lane 1 as the negative control. (B) The transcriptions of four *pilA*_Sa_ genes (from top to bottom) in *S* *. aurantiaca* DSM17044 vegetative cells were determined with the RT-PCR using specific primers (listed in Table 1). Lanes 1~3 show the agarose gel electrophoresis of RT-PCR products using total RNA, genomic DNA and cDNA as the template, respectively.

After identifying multiple type IV pilin homologues in *S* *. aurantiaca* DSM17044, we sought to determine their potential biological functions. A western blot using an anti-PilA_Mx_ antibody was employed to investigate pilin levels in whole cells and surface components of *S* *. aurantiaca* DSM17044. As shown in Figure 3A, positive immuno-blot signals were observed in both lanes loaded with whole cell lysates and with isolated extracellular components. This result indicates that the polyclonal anti-PilA_Mx_ antibody recognizes the pilin protein from *S* *. aurantiaca* DSM17044, which might be due to the similarities between PilA_Mx_ and PilA_Sa_ proteins (Figure 1). Furthermore, the results show that at least one of the PilA_Sa_ proteins was expressed in *S* *. aurantiaca* DSM17044 both intracellularly and extracellularly. Next, the transcription levels of the four *pilA*_Sa_ genes in *S* *. aurantiaca* DSM17044 were determined using RT-PCR. The results show that the mRNA all of four *pilA*_Sa_ genes could be detected in *S* *. aurantiaca* DSM17044 cells during vegetative growth (Figure 3B).

**Figure 4:**
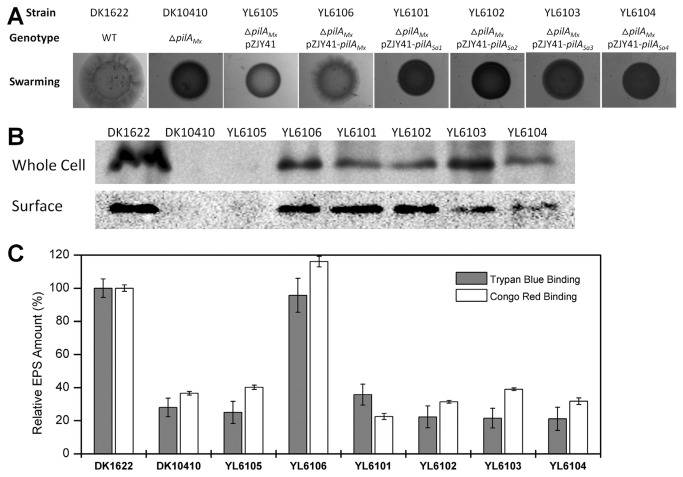
Effects of heterologously expressed *pilA*_*Sa*_ genes in *M. xanthus* DK10410 on S-motility ability, TFP biogenesis and EPS production. (A) S-motility and surface pili of different *M. xanthus* strains. Top to bottom rows show swarming on 0.3% CTT agar surfaces after 120 h incubation. (B) Whole-cell pilin (upper row) and surface pili (bottom row) of *M. xanthus* cells were tested using western-blot probed by anti-PilA_Mx_ antibody. (C) Quantitative analysis of EPS production in different *M. xanthus* strains using trypan blue binding assay (grey columns) and congo red binding assay (white columns). Values for all strains were normalized to the wild-type DK1622, respectively. The data represent triplicate experiments, and mean ± SD is plotted.

The difficulties of genetic manipulation hindered a deeper investigation of PilA_Sa_ in *S* *. aurantiaca* DSM17044; therefore, the *pilA*_Sa_ genes were transferred into *M. xanthus* DK10410 (Δ*pilA*_Mx_) using the *E. coli-M. xanthus* shuttle vector pZJY41 [32]. To prevent the potential influence of upstream sequences, the promoter and signal peptide-coding region of each *pilA*_Sa_ gene was replaced by its *pilA*_Mx_ counterpart. The S-motilities of the *M. xanthus* strains were assayed on CTT medium containing 0.3% agar. As shown in Figure 4A, strains YL6101~4 carrying the *pilA*_Sa1~4_ genes exhibited deficient S-motilities and had smooth colony edges, while strain YL6106 (Δ*pilA*_Mx_, pZJY41-*pilA*_Mx_), the positive control, showed normal S-motility on soft agar and phenotypically resembled wild-type DK1622. The whole cellular and extracellular components of these *M. xanthus* cells were probed by western-blot using an anti-PilA_Mx_ antibody, and positive bands were revealed in all of the samples from YL6101~4 (Figure 4B). These results suggest that although the *pilA*_Sa1~4_ genes from *S* *. aurantiaca* DSM17044 are expressed by *M. xanthus* DK10410 (Δ*pilA*_Mx_), this does not restore S-motility on a soft agar surface. Therefore, EPS production was examined in these strains, which is another key component for S-motility in addition to TFP [11].

Previous studies have shown that the surface pilus (extracellular PilA) is the positive regulator of EPS production in *M. xanthus* [16]. As shown in Figure 4C, complementary strain YL6106 containing the *pilA*_Mx_ gene in a Δ*pilA*_Mx_ fully restored EPS production to levels observed in the wild type DK1622, while the EPS levels in strains YL6101~4 (carrying *pilA*_Sa1~4_ genes, respectively) were significantly lower (60~80%) than that of the wild-type strain DK1622 and similar to that of strain DK10410 (Δ*pilA*_Mx_). This result shows that the presence of the extracellular PilA_Sa_ did not up-regulate EPS production in *M. xanthus*. Meanwhile, several pieces of evidence have shown that PilA_Mx_ specifically recognizes and interacts with the EPS of *M. xanthus* [11,17,31], and that EPS is the trigger for TFP retraction, which enables *M. xanthus* cells to perform S-motility on agar [11]. Therefore, we hypothesized that the lack of S-motility in DK10410 (Δ*pilA*_Mx_) carrying the *pilA*_Sa_ genes might be due to deficient EPS production or failure of the PilA_Sa_ proteins to recognize the EPS of *M. xanthus.*

### *M. xanthus* cells carrying *pilA*_Sa2_ demonstrated reduced TFP-dependent motility in 1% methylcellulose solution

**Figure 5:**
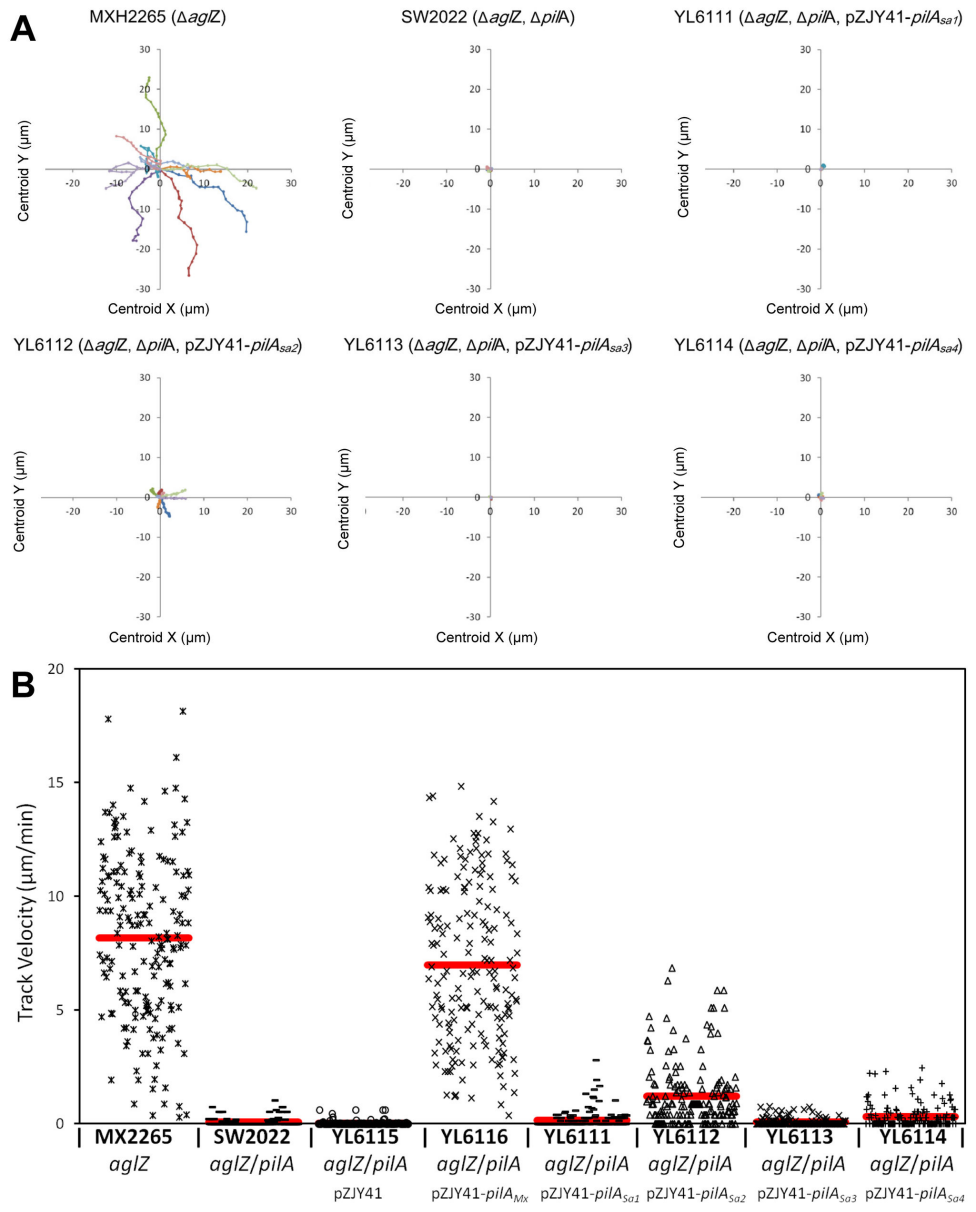
Tracking motility of *M. xanthus* strains containing *pilA*_*Sa*_ genes in 1% methycellulose solution. Different *M. xanthus* cells were submurged in 1% methylcellulose solution and cell movements were recorded by time lapse photography. Motility and trajectories of 10 isolated cells were analyzed. Data are presented as tracking plots (panel A) and as diagrams (panel B). In panel A, a static synthetic view of cell motility tracks was generated as described in the *Materials and Methods*, and one color was applied for each trajectory. In panel B, the red lines show the average velocitis of respective strains.

Next, *M. xanthus* cells were analyzed for motility on a polystyrene surface submerged in a methylcellulose solution because it has been proposed that *M. xanthus* cells could bypass the need for EPS to anchor their TFP and conduct TFP-dependent single-cell motility under this condition [14]. The *aglZ* gene was in-frame deleted in strains YL6101~6 to generate strains YL6111~6 (Table 2), respectively, which inactivated the adventurous motility (A-motility) [33] in these strains to eliminate potential motile backgrounds [10]. As shown in Figure 5, and in agreement with previous findings [14], MXH2265 (Δ*aglZ*) cells and YL6116 cells (containing the *pilA*_Mx_ gene in a Δ*aglZ* and Δ*pilA*_Mx_ mutant background) exhibited similar levels of single-cell motility in the methylcellulose solution, while active motility was totally eliminated in the respective mutant strains defective in surface pilus biogenesis, i.e., SW2022 (Δ*aglZ*, Δ*pilA*_Mx_) and YL6115 (Δ*aglZ*, Δ*pilA*_Mx_, pZJY41). Of the four strains carrying *pilA*_Sa1~4_ genes, the YL6112 (ΔaglZ, Δ*pilA*_Mx_, pZJY41-*pilA*_Sa2_) cells showed relatively active single-cell motility, which was significantly different from the YL6111, YL6113 and YL6114 cells (carrying *pilA*_Sa1_, *pilA*_Sa3_ and *pilA*_Sa4_, respectively), although at a reduced level compared with that of MXH2265 (Δ*aglZ*) cells.

**Table 2:** Bacterial strains and plasmids used in this study.

**Designation**	**Relevant Feature**	**Ref. or Source**
**Strain**		
*M. xanthus*		
DK1622	Wild type	
DK10410	DK1622, *ΔpilA*, missing PilA	[41]
MXH2265	DK1622, *ΔaglZ*, deficient in A-motility	[33]
SW2022	DK1622, *ΔaglZ*, *ΔpilA*	[14]
YL6101	DK1622, *ΔpilA*, containing pTZG-1	This study
YL6102	DK1622, *ΔpilA*, containing pTZG-2	This study
YL6103	DK1622, *ΔpilA*, containing pTZG-3	This study
YL6104	DK1622, *ΔpilA*, containing pTZG-4	This study
YL6105	DK1622, *ΔpilA*, containing pZJY41	This study
YL6106	DK1622, *ΔpilA*, containing pTZG-5	This study
YL6111	DK1622, *ΔaglZ*, *ΔpilA*, containing pTZG-1	This study
YL6112	DK1622, *ΔaglZ*, *ΔpilA*, containing pTZG-2	This study
YL6113	DK1622, *ΔaglZ*, *ΔpilA*, containing pTZG-3	This study
YL6114	DK1622, *ΔaglZ*, *ΔpilA*, containing pTZG-4	This study
YL6115	DK1622, *ΔaglZ*, *ΔpilA*, containing pZJY41	This study
YL6116	DK1622, *ΔaglZ*, *ΔpilA*, containing pTZG-5	This study
*S* *. aurantiaca*		
DSM17044	Type strain for *S* *. aurantiaca* , ATCC 25190	[24]
*E. coli*		
DH5α	Host for cloning	[61]
**Plasmid**		
pZJY41	Shuttle vector in *E. coli*-*M. xanthus*; Kan^r^ Amp^r^	[32]
pTZG-1	PSP_Mx_ and *pilA*_Sa1_ fusion fragment in pZJY41*	This study
pTZG-2	PSP_Mx_ and *pilA*_Sa2_ fusion fragment in pZJY41	This study
pTZG-3	PSP_Mx_ and *pilA*_Sa3_ fusion fragment in pZJY41	This study
pTZG-4	PSP_Mx_ and *pilA*_Sa4_ fusion fragment in pZJY41	This study
pTZG-5	PSP_Mx_ and *pilA*_Mx_ fusion fragment in pZJY41*	This study

* *pilA*_Mx_ stands for *pilA* in *M. xanthus* DK1622, PSP_Mx_ stands for promoter and signal peptide sequence of *pilA* gene in *M. xanthus* DK1622, and *pilA*_Sa_ stands for *pilA* in *S* *. aurantiaca* DSM17044.

**Figure 6:**
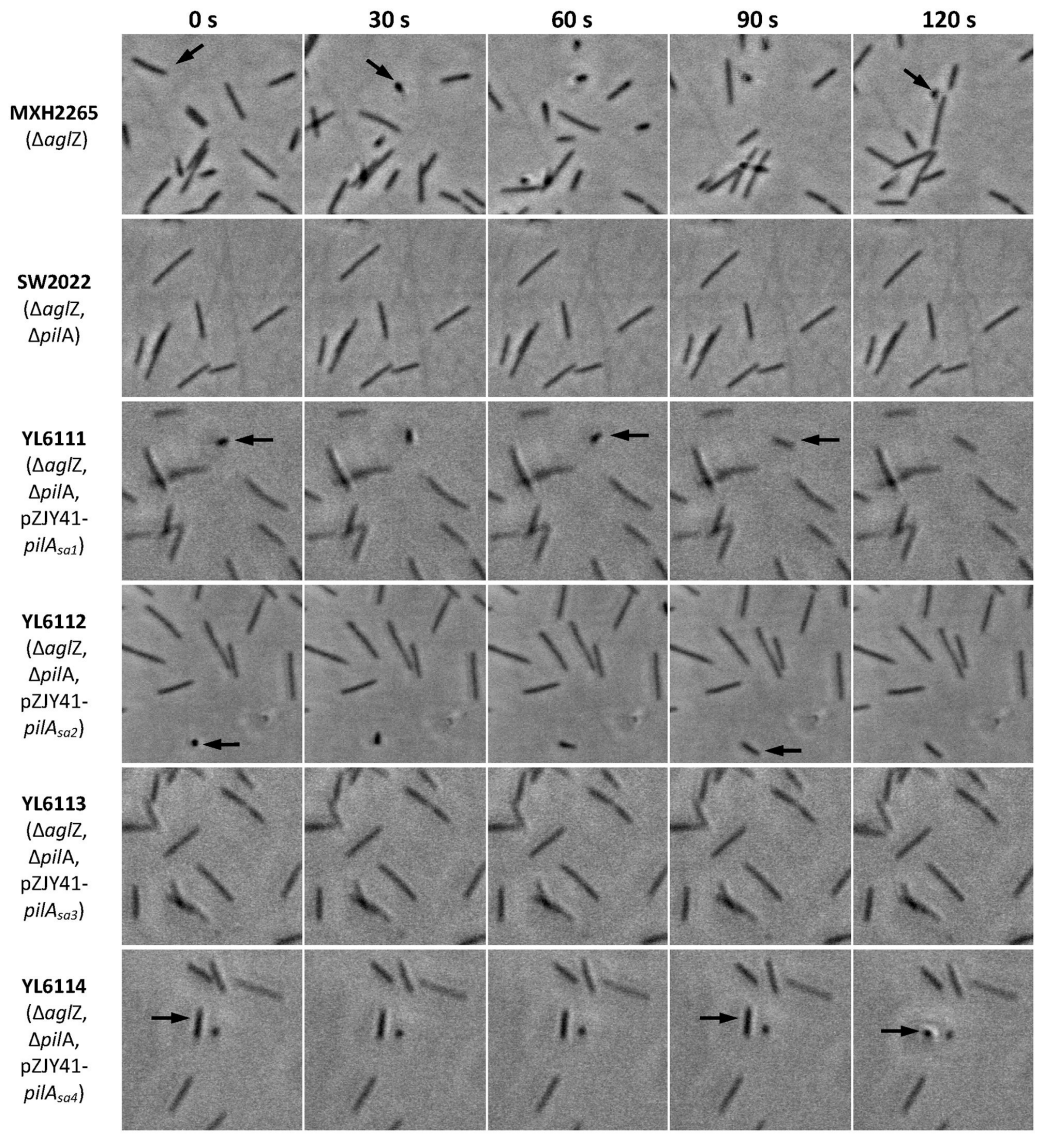
Tethering behavior of *M. xanthus* cells containing *pilA*_*Sa*_ genes in 1% methycellulose solution. *M. xanthus* cells were deposited onto polystyrene plates and submerged 1% methylcellulose solution, and individual cells were analysed for the tethering behaviour on solid surfaces. Tethered cells appear as dots in the image, indicated by black arrows. Left to right images were taken at 30 s intervals.

While *pilA*_Sa1~4_ genes from *S* *. aurantiaca* DSM17044 were all extracellularly expressed in *M. xanthus* cells (Figure 4B), only the cells carrying *pilA*_Sa2_ exhibited reduced motility in methylcellulose (Figure 5), which might be due to differences in the TFP retraction ability of these cells. To further test this possibility, the tethering behavior [10,14] of *M. xanthus* cells was investigated in the methylcellulose solution. As shown in Figure 6, the motile cells of MXH2265 (Δ*aglZ*) and YL6112 (*ΔaglZ*, Δ*pilA*_Mx_, pZJY41-*pilA*_Sa2_) were occasionally tethered to the surface with their TFP, resulting in the detection of cells with one end attached to the solid surface and lifted-up cell bodies. Cells lacking TFP, e.g., SW2002 (Δ*aglZ*, Δ*pilA*_Mx_), were non-motile and unable to tether. The YL6111 cells (*ΔaglZ*, Δ*pilA*_Mx_, pZJY41-*pilA*_Sa1_) and YL6114 cells (*ΔaglZ*, Δ*pilA*_Mx_, pZJY41-*pilA*_Sa4_) were not motile while exhibiting occasional tethering behavior, which is similar to the phenotype of the TFP retraction-deficient mutant Δ*pilT* [10]. This indicates that YL6111 and YL6114 produced stable surface TFP that allow the cells to tether but the pili are unable to retract. As a consequence, S-motility on agar or in methylcellulose is entirely impaired in these two strains (Figures 4A and 5). Interestingly, YL6113 cells (*ΔaglZ*, Δ*pilA*_Mx_, pZJY41-*pilA*_Sa3_) showed no motility or tethering in methylcellulose solution (Figure 6), which implies that these cells lack stable surface pili.

### Expression of *pilA*_Sa_ genes affected developmental abilities of *M. xanthus* host cells

**Figure 7:**
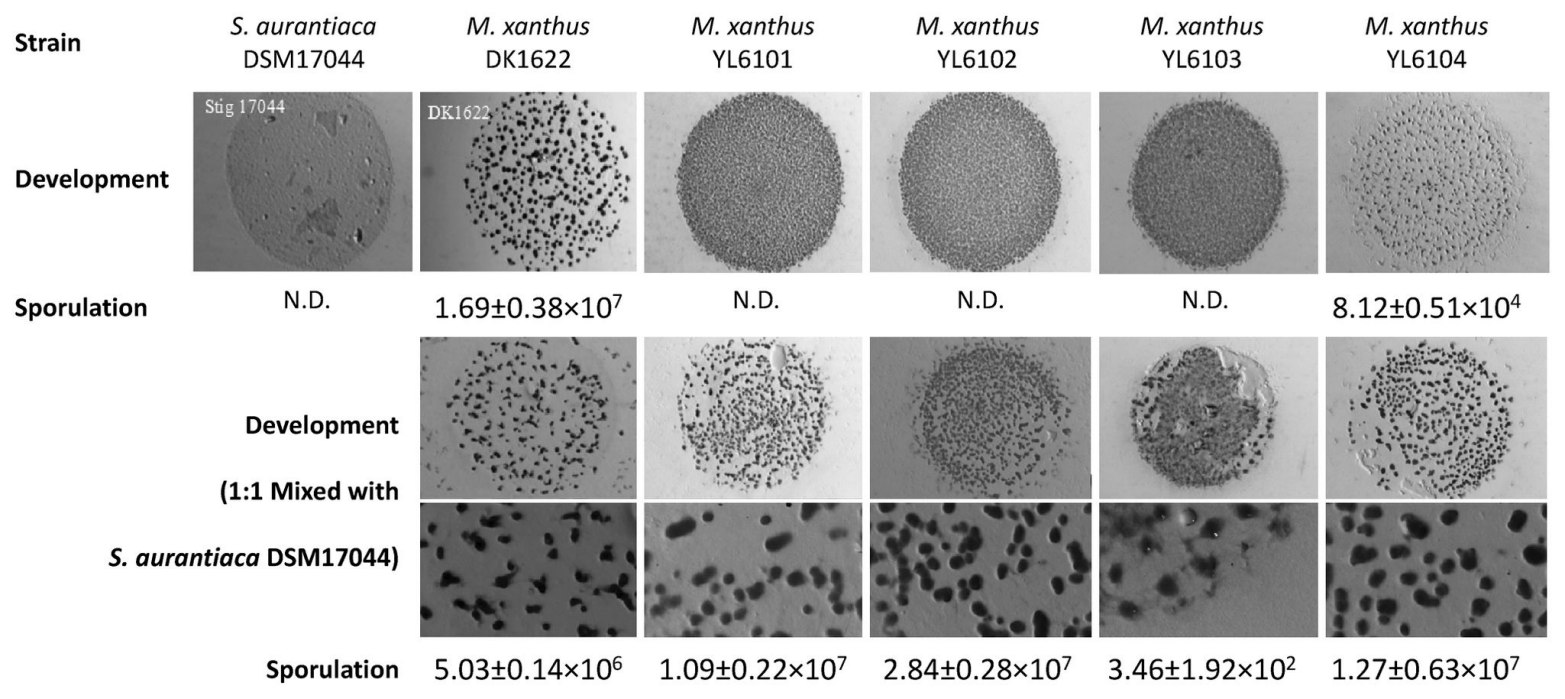
Phenotypes of fruiting body formation and sporulation. Fruiting body formation (1^st^ row) and sporulation (2^nd^ row) of the *S* *. aurantiaca* strain DSM17044 and the *M. xanthus* strains DK1622 (wild-type), YL6101 (*ΔpilA*, pZJY41-*pilA*_Sa1_), YL6102 (*ΔpilA*, pZJY41-*pilA*_Sa2_), YL6103 (*ΔpilA*, pZJY41-*pilA*_Sa3_) and YL6104 (*ΔpilA*, pZJY41-*pilA*_Sa4_) were assayed after incubation of 5.0×10^6^ vegetative cells for 5 d on TPM agar. 2.5×10^6^ cells of *S* *. aurantiaca* DSM17044 were pre-mixed with 2.5×10^6^ cells of different *M. xanthus* strains, respectively, and fruiting body formation (3^rd^ and 4^th^ row) and sporulation (5^th^ row) of the mixing cultures were assayed on TPM agar after 5 d incubation. The images in 4^th^ row exhibit a magified protion of the images in 3^rd^ row, respectively. ‘N.D.’ represents ‘not detected’.

Because it has been shown that the deletion or mutation of *pilA*_Mx_ compromise the fruiting body formation of *M. xanthus* on TPM agar [17,34], we wondered if the expression of *pilA*_Sa_ genes could affect the development of their host *M. xanthus* cells. As shown in Figure 7 (upper row images), after being incubated on TPM agar for 5 days, YL6101, YL6102 and YL6103 (Δ*pilA*_Mx_ and carrying *pilA*_Sa1_, *pilA*_Sa2_ and *pilA*_Sa3_, respectively) formed immature fruiting bodies and were all deficient in myxospore production. However, YL6104 (Δ*pilA*_Mx_, pZJY41-*pilA*_Sa4_) was phenotypically similar to wild type DK1622, exhibiting normal fruiting body formation and reduced sporulation. While *S* *. aurantiaca* DSM17044 did not form fruiting bodies on TPM agar, mixing DSM17044 cells with *M. xanthus* cells significantly affected the development of the latter (Figure 7, images in bottom two rows). The fruiting body formation and sporulation of YL6101, YL6102 and YL6104 (Δ*pilA*_Mx_ and carrying *pilA*_Sa1_, *pilA*_Sa2_ and *pilA*_Sa4_, respectively) were fully restored compared to those of wild type DK1622 after 1:1 mixing with *S* *. aurantiaca* DSM17044 cells. As for YL61103 (Δ*pilA*_Mx_, pZJY41-*pilA*_Sa3_), these abilities were partially complemented after mixing. Considering *Stigmatella* has complicated and specific fruiting body structures, which are morphologically different from the round *Myxococcus* fruiting bodies [2,20], the fruiting bodies on the mixing plates were most likely formed by the *M. xanthus* cells rather than the *S* *. aurantiaca* DSM17044 cells.

## Discussion

In this study, four genes encoding type IV pilin homologues were identified in *S* *. aurantiaca* DSM17044 (Figure 1), all of which were transcribed during vegetative growth, and at least one of these genes was expressed in DSM17044 both intracellularly and extracellularly (Figure 3). Moreover, there are five *pilA* homologues in *S* *. aurantiaca* DW4/3-1 [20], two *pilA* homologues in *M. xanthus* DK1622 [35], and four *pilA* homologues in *S. cellulosum* so ce56 [36], which is consistent with the finding that gene duplicates are common in the genomic sequence of myxobacterial strains as a result of gene diversion and duplication [20,35,36,37]. Some duplicated genes result in a similar protein product, i.e., two genes (*MXAN_5430* and *MXAN_5432*) encode protein S in the *M. xanthus* DK1622 genome [38], which are assumed to accelerate the biosynthesis of protein S and the formation of myxospores during fruiting body development [39]. Some duplications are assumed to be followed by divergence of the new gene copies, endowing them with new specificities [35]. For example, two copies of the chaperone *groEL* gene are present in the *M. xanthus* DK1622 genome; *groEL1* (*MXAN_4895*) is more active in cellular development and sporulation, while *groEL2* (*MXAN_4467*) is important for predation behavior [40]. As for the *pilA* genes in myxobacteria, the significance of gene duplication remains unclear. In *M. xanthus* DK1622, the *pilA*_Mx_ gene encodes the type IV pilin and is responsible for TFP assembly and S-motility [41], while the function of *MXAN_5675* (annotated as fimbrial protein) is still unknown.

To determine their potential biological functions, the *pilA*_Sa_ genes from *S* *. aurantiaca* DSM17044 were transferred into *M. xanthus* DK10410 (Δ*pilA*_Mx_) and were successfully extracellularly expressed, which might be because the promoter and signal peptide-coding region of the *pilA*_Mx_ gene was inserted in front of each *pilA*_Sa_ gene in every construct. In bacteria, the pilin protein is synthesized as prepilin with an N-terminal hydrophilic signal peptide that is recognized and cleaved by the prepilin peptidase PilD [42]. A previous study has shown that deletion or mutation of the *pilA*_Mx_ signal peptide significantly compromises PilA_Mx_ processing and production [17]; therefore, the whole *pilA*_Mx_ signal peptide was stitched to each *pilA*_Sa_ to ensure the gene product could be processed correctly in its *M. xanthus* host. In addition to the processing, mature pilin proteins are assembled into polar filaments mediated by the PilB ATPase [13,15], which is a key step in pilin protein secretion. Our results suggests that despite the differences in amino acid sequences and predicted protein structures of the PilA proteins (Figure 1), all four PilA_Sa_ proteins could be exported extracellularly by the PilB ATPase (Figure 4B), indicating that the substrate specificity of PilB in *M. xanthus* is relatively low.

According to their various motility-related phenotypes (Figure 4~6), the *M. xanthus* strains carrying different *pilA*_Sa_ genes were categorized into three distinct types. The type I strains (YL6101 carrying *pilA*_Sa1_ and YL6104 carrying *pilA*_Sa4_) produced stable surface pili (detected by both western blot and the tethering assay), but were not motile on soft agar or in methylcellulose solution, which indicated that their TFP_Sa_ were unable to retract. The type II strain (YL6102 carrying *pilA*_Sa2_) also produced stable surface pili and did not display S-motility on soft agar. However, cells in this category showed single-cell motility in methylcellulose solution, albeit at a reduced level compared with the motility of cells carrying *pilA*_Mx_. Therefore, it was suggested that *M. xanthus* cells carrying *pilA*_Sa2_ produced retractable TFP_Sa2_ and can perform TFP-dependent motility in the methylcellulose solution, and the nonspecific interactions of TFP_Sa2_ with the polystyrene surface in the methylcellulose solution might compensate for the absence of the TFP_Sa2_-EPS specific interaction. Previous studies showed that swarms of *M. xanthus* and *S* *. aurantiaca* initially merged on an agar surface but subsequently separated and established separate fruiting bodies [43], which implies a potential specific recognition of self-EPS components by the motility systems of these two species during the swarming and development process. The type III strain (YL6103 carrying *pilA*_Sa3_) did not exhibit motility or tethering behaviors, indicating that they produced unstable surface pili, which might be attributed to the unique straight α-helical domain of PilA_Sa3_ (Figure 1B). The curved structure of the PilA_Mx_ α-helical domain has been shown to be essential for stable pili production, and the formation of a kink in the α-N-terminal subdomain has been implicated as in assisting in the tight packing of pilin subunits into TFP [29,44]. In the predicted structure of PilA_Sa3_, this kink was missing due to unique residues at positions 22~27 in its primary structure. We also noticed that both counterparts for *pilA*_Sa2_ and *pilA*_Sa3_ (*STAUR_6449* and *6450*) were located in a gene cluster in *S* *. aurantiaca* DW4/3-1 genome, which is predicted to produce TFP components (from *STAUR_6441* to *STAUR_6458*). It has been shown that the pilin gene in the TFP gene cluster normally encodes the functional type IV pilin for twitching or social motility, e.g., *pilA*_Mx_ in *M. xanthus*, *pilA* in *P. aeruginosa* and *pilE* in *N. gonorrhoeae* [45]. In *S* *. aurantiaca* , we propose that *pilA*_Sa2_ rather than *pilA*_Sa3_ could be responsible for the type IV pilin production to perform group motility.

*S* *. aurantiaca* is well known for its complicated and particular fruiting body [2,20], which is quite different from the one formed by *Myxococcus* cells. However, it has been shown that the expression profile of the development-specific genes in these two species is extramely similar. In particular, the genes involved in signal transduction pathways that are important for fruiting body formation in *M. xanthus* are conserved in *S* *. aurantiaca* [20]. In *M. xanthus*, the PilA_Mx_ protein is thought to be involved in the fruiting body formation process. The deletion of *pilA*_Mx_ compromises the fruiting body formation of *M. xanthus* on TPM agar [34], which may be because surface pili serve as a sensor to provide signals to the Dif chemosensory pathway, thereby controlling EPS production [16]. Moreover, a mutation in the PilA_Mx_ protein has been shown to diminish the fruiting body formation of *M. xanthus* by leading to an accumulation of PilA_Mx_ in the periplasmic space and reducing surface EPS production [17]. Expression of *pilA*_Sa4_ in a *M. xanthus* Δ*pilA*_Mx_ background (strain YL6104) phenotypically restored the fruiting body formation and reduced sporulation compared to levels of wild-type DK1622 cells (Figure 7), while YL6104 cells produced a similar amount of EPS compared to DK10410 (Δ*pilA*_Mx_) cells (Figure 4C). This suggested that the PilA_Sa4_ protein might positively regulate the fruiting body formation of *M. xanthus* cells through an unknown mechanism rather than by regulating of EPS production. More interestingly, after being mixed with the *S* *. aurantiaca* DSM17044 cells, the *M. xanthus* cells with stable exogenous TFP_Sa_, i.e., cells of YL6101, YL6102 and YL6104, could form mature fruiting bodies and produce wild-type levels of myxospores (Figure 7). Because the specific interaction between TFP and EPS has been suggested in *M. xanthus* [11,17,31], we favor the hypothesis that TFP_Sa_ recognize the EPS from *S* *. aurantiaca* and up-regulate the developmental process of the *M. xanthus* cells. We are currently addressing this hypothesis by examining interactions of PilA_Sa_ proteins with EPS from *M. xanthus* and *S* *. aurantiaca* .

## Materials and Methods

### Bacterial strains and cultural conditions

Bacterial stains used in this study were listed in Table 2. *M. xanthus* cells were grown in CTT medium [46] at 32°C, and *S* *. aurantiaca* cells were cultured in VY/2 medium [47] at 32°C. The developmental assay of myxobacterial cells was performed on TPM plates [48]. The S-motility assay was conducted on CTT plates containing 0.3% agar [49]. *E. coli* cells were cultured in Luria-Bertani (LB) medium [50] at 37°C. When necessary, kanamycin (Kan) was added to the medium to a final concentration of 40 µg/ml.

### Amplification of the *S* *. aurantiaca* DSM17044 genes homologous to *pilA* by polymerase chain reaction (PCR)

Five sets of specific primers (Table 1) were designed according to the sequences of the five *pilA* homologues in the *S* *. aurantiaca* strain DW4/3-1 genome [20], and were used in the subsequent PCR with DSM17044 genomic DNA as the template. The DSM17044 genomic DNA was isolated and purified as described previously [51]. For PCR, a 50 µl-volume reaction solution was prepared by mixing 1 µl of template DNA (20 ng/µl), 1 µl of each primer (50 µM), 4 µl of dNTPs (2.5 mM), 1 µl of pfu DNA polymerase (2.5 U/µl, Fermentas), 25 µl of 2×GC Buffer I (Takara Bio) and 17 µl of ddH_2_O. The conditions for the PCR amplification were as follows: the initial denaturation step was at 94°C for 3 min, annealing was at 65°C for 1 min, polymerization was at 72°C for 1 min, subsequent denaturation was at 94°C for 1 min, and there were 30 cycles. The PCR products were purified with the EZNA Cycle pure kit (Omega). Four genes were amplified from DSM17044 genomic DNA using the primer sets targeting genes *STAUR_0004*, *6449*, *6450* and *6924* in the DW4/3-1 genome (Table 1), which were referred to as the *pilA*_Sa1_, *pilA*_Sa2_, *pilA*_Sa3_ and *pilA*_Sa4_ genes in this study (*pilA* in *Stigmatella* *aurantiaca* DSM17044), respectively.

The purified fragments of the *pilA*_Sa_ genes were ligated into the pGEM-T Easy vector (Promega), electroporated into *E. coli* DH5α, and the recombinant transformants were screened according to the standard protocol [50]. The recombined plasmids with a proper insertion were extracted and sequenced. The sequences of the four *pilA*_Sa_ genes (*pilA*_Sa1~4_) were deposited in the GenBank database (www.ncbi.nlm.nih.gov) with accession number KF113889, KF113890, KF113891 and KF113892, respectively.

### Bioinformatic analysis

The amino acid sequences of PilA in *M. xanthus* DK1622 (referred to as PilA_Mx_) and PilA_Sa1~4_ were compared and aligned using the ClustalX program version 1.83 [52]. The amino acid sequences of the PilA proteins from different myxobacterial strains were retrieved from the Genbank database, and the phylogenetic reconstruction of the sequences was conducted using distance/neighbor joining programs with the Poisson correction distance model in MEGA software package version 4.0 [53]. The interior branch length supports were from 1000 replicates. The putative 3D structures of PilA_Mx_ and PilA_Sa1~4_ were constructed on-line using 3D-JIGSAW (http://bmm.icnet.uk/~3djigsaw/) [51] and further confirmed by Swiss-Model (http://swissmodel.expasy.org/).

### Reverse transcription polymerase chain reaction (RT-PCR)

Total RNA of *S* *. aurantiaca* DSM17044 was extracted using the SV total RNA isolation kit (Promega), and the genomic DNA was removed with the DNA free kit (ABI) following the protocols recommended by the manufacturers. RT-PCR was performed as described previously [54]. The complimentary DNA (cDNA) was synthesized using the downstream primer (RT-R primer, Table 1), and the double stranded DNA was amplified with the proper primer pair (RT-F and RT-R primers, Table 1) for each *pilA*_Sa_ gene.

### Construction and transformation of the *pilA*-containing plasmids

The promoter and signal peptide fragment of *pilA*_Mx_ (referred as to PSP_Mx_) was amplified using primers DK *pilA*-SP-F and DK *pilA*-SP-R (Table 1) and using *M. xanthus* genomic DNA as a template. The PSP_Mx_ fragment was stitched onto each *pilA*_Sa_ gene through over-lap PCR as described previously [12]. For the over-lap PCR, DK *pilA*-F and Stig *pilA*-R (Table 1) were used as primers, and the fragments of *pilA*_Sa_ and PSP_Mx_ were used as templates. The PSP_Mx_ and *pilA*_Mx_ fusion fragment was directly amplified from *M. xanthus* genomic DNA using the DK *pilA*-SP-F and DK *pilA*-R (Table 1) primers. After purification, the fusion products were ligated into EcoRV-digested plasmid pZJY41 as previously described [32], resulting in the recombinant plasmids pTZG-1~5 (Table 2), which were subsequently transferred into *E. coli* DH5α and sequenced. The *pilA*-containing plasmids pTZG-1~5 and empty plasmid pZJY41 were, respectively, electroporated into *M. xanthus* DK10410 (Δ*pilA*) or SW2002 (Δ*aglZ*, Δ*pilA*) according to the standard protocol [55]. After 7 days, transformants were selected from CTT plates containing 40 µg/ml Kan. The positive transformants were purified, and the plasmids were extracted for confirmation as previously described [32].

### S-motility assay

S-motility of *M. xanthus* cells on agar surfaces was analyzed as described previously [49]. Cells in mid-log phase were collected from CTT broth by centrifugation and resuspended in CTT medium to a final concentration of 5×10^9^ cells/ml. Aliquots of a 2 µl cell suspension were spotted onto swarm plates (CTT medium containing 0.3% agar) and incubated at 32°C for 5 days before record.

### Immunoblot analysis of pilin proteins

Cell-surface pili of *M. xanthus* or *S* *. aurantiaca* DSM17044 were isolated from 10^10^ cells as previously described [41]. Isolated pili were resuspended in SDS-PAGE loading buffer and boiled for 10 min. For whole-cell lysates, 10^8^ *M. xanthus* or *S* *. aurantiaca* DSM17044 cells were directly lysed by boiling in SDS-PAGE loading buffer for 10 min. The samples were then separated by SDS-PAGE (10% gel) and subjected to western-blot analysis using standard methods [56]. Primary anti-PilA_Mx_ antibody [12] was used at a 1:4000 dilution, goat anti-rabbit horseradish peroxidase conjugated secondary antibody (Pierce) was used at a 1:4000 dilution. The blots were developed, and the bands were detected using the ECL Chemiluminescence kit (Tiangen).

### Examination of extracellular polysaccharides (EPS) production

Two quantitative methods were used to examine EPS production of *M. xanthus* cells, namely the congo red binding assay [57] and the trypan blue binding assay [16,58]. All strains tested were harvested from CTT broth at the mid-log growth phase and resuspended in MOPS buffer (10 mM MOPS, 8 mM MgSO_4_, pH 7.6) to a concentration of 5 × 10^8^ cell/ml. The EPS production of all strains was normalized to that of the wild-type strain DK1622, which was arbitrarily set to 1. Experiments were performed in triplicate.

### Methylcellulose assay for TFP-dependent motility

The TFP-dependent motility of *M. xanthus* cells was analyzed using a previously published protocol [10,14]. Polystyrene plates (Costar^TM^ cell culture plates, Fisher) were used as a testing surface. Cell movements were monitored with a Nikon Eclipse TE2000-S inverted microscope through a 40× objective, captured with a Nikon DXM1200F CCD camera and recorded with Nikon ACT-1 software (Version 2.62). Continuous images were taken at 10 s intervals and stored as TIFF image sequence files. The velocity measurements and trajectory tracking were performed as previously described [14] using Manual Tracking [59], a plugin for the ImageJ software (http://rsb.info.nih.gov/ij/). A static synthetic view of cell motility tracks was generated and the recorded coordinates were exported to Microsoft Excel to present the data as plots. The tethering behavior of *M. xanthus* cells was recorded and analyzed in the same experimental system as previously described [10,14]. When deposited in 1% methylcellulose medium, some wild-type *M. xanthus* cells were observed to be perpendicular to the polystyrene surface, and appeared to have one of their cell ends tethered to the solid surface with the TFP. Cells with unretractable surface TFP (*ΔpilT*) were non-motile in this assay while able to be tethered [10], and cells lacking TFP (*ΔpilA*) or stable surface TFP (SW2031, *pilA*-A32V) were non-motile and unable to be tethered [44]. The tethered cells were identified in a series of images as those with one end of the cell attached to the solid surface and lifted-up cell bodies.

### Development assays

*M. xanthus* cells were grown in CTT to mid-log phase and concentrated to 5×10^9^ cells/ml in TPM buffer (10 mM Tris-HCl, 1 mM KH_2_PO_4_, 8 mM MgSO_4_, pH 7.6). Ten microliter aliquots of concentrated cells were spotted onto TPM agar and incubated for 5 days at 32°C [60]. Pictures of fruiting body were taken using a Nikon SMZ1500 dissection microscope and recorded by Nikon ACT-1 software (Version 2.62).

For the mixing development experiments, *S* *. aurantiaca* DSM17044 cells were grown in VY/2 to mid-log phase and concentrated to 5×10^9^ cells/ml in TPM buffer. The cell suspension of *S* *. aurantiaca* DSM17044 was mixed with an equal volume of various *M. xanthus* cells suspension (5×10^9^ cells/ml) to prepare the mixed inoculums, and 10 µl aliquot of the mixed cells were spotted onto TPM agar and incubated for 5 days at 32°C. The development was recorded as described above.

Sporulation was determined as previously described [60] with minor modifications. The 5-day cultured fruiting bodies were scraped from TPM agar, resuspended in 200 µl of TPM buffer and homogenized by slight sonication. The suspension was incubated at 50°C for 2 hours, serially diluted, mixed with CTT media containing 0.3% agar, poured onto CTT plates with 1.5% agar, and incubated at 32°C for 5 days. The sporulation efficiencies were calculated as the number of colonies that appeared on the CTT plates relative to the original number of cells spotted. Three replicate experiments were performed.
